# Silicified Wood with Dual Fire Retardancy and Thermal Management Functionalities

**DOI:** 10.3390/polym17172293

**Published:** 2025-08-25

**Authors:** Kunkun Tu, Jinjing Liu, Jiayi Li, Suhao Li, Xu Zhang, Shihang Li

**Affiliations:** 1 Jiangsu Key Laboratory of Coal-Based Greenhouse Gas Control and Utilization, China University of Mining and Technology, Xuzhou 221008, China; liujinjing@cumt.edu.cn (J.L.); jiayili@cumt.edu.cn (J.L.); ts23160124p31@cumt.edu.cn (X.Z.); 2 Carbon Neutrality Institute, China University of Mining and Technology, Xuzhou 221008, China; 3 School of Environment Science and Spatial Informatics, China University of Mining and Technology, Xuzhou 221116, China; 4 School of Safety Engineering, China University of Mining and Technology, Xuzhou 221116, China; lisuhao2005@126.com

**Keywords:** silicification, SiO_2_@wood, fire retardancy, thermal management, energy-efficient buildings

## Abstract

Fire retardancy and thermal management are critical for energy-efficient, fire-safe buildings. Natural wood, a mainstream construction material, possesses inherent advantages but lacks such dual functionality. Silicified wood was fabricated via multi-cycle silicification of native wood, where SiO_2_ uniformly infiltrates and fills the lumens. The treated wood material displays an improved limiting oxygen index (LOI) from 21.9% to 36.0%, and delayed ignition from 15 s to 50 s, compared to untreated wood. It demonstrates a low thermal conductivity of 0.074 W·m^−1^·K^−1^, showing enhanced emissivity. When heated on a 75 °C hot plate, the silicified wood surface reaches ~50 °C after 5 s, versus ~60 °C for native wood. These enhancements collectively improve thermal management performance, achieving insulation through reduced thermal conduction and passive cooling via optimized infrared regulation. Ultimate tensile stress remains nearly unchanged post-treatment, while toughness is significantly improved. This work advances wood as a sustainable building material, with promising potential for fire-safe, energy-efficient construction applications.

## 1. Introduction

Building materials integrating flame retardancy and thermal management are critical for energy-efficient and fire-safe construction, as they reduce energy consumption for temperature regulation while mitigating fire risks [1,2,3]. Among various candidates, natural wood stands out for its inherent advantages: as a renewable, carbon storage material with a hierarchical porous structure, it exhibits intrinsic thermal insulation potential, and its abundance makes it a staple in traditional and modern buildings [4,5,6]. These attributes position wood as a promising substrate for developing multifunctional construction materials, bridging sustainability and performance [7]. However, natural wood has inherent limitations that hinder its full application in high-performance construction: its intrinsic flammability presents a major limitation, posing significant fire safety risks in practical use, which has spurred extensive efforts to reduce flammability [8,9]. Furthermore, although natural wood possesses certain thermal management capabilities, its native performance remains inadequate to fully meet the stringent energy-saving requirements of modern buildings, necessitating further enhancement to reduce building energy consumption effectively [10].

In recent years, advanced wood modification techniques have emerged rapidly. Flame-retardant modification of wood is primarily achieved through the integration of functional flame retardants with treatment processes such as impregnation and surface modification [11]. Wood thermal management, encompassing thermal insulation and passive cooling, involves strategies including delignification, incorporation of phase change materials, and micro/nanostructure regulation [12,13]. Notably, cellulose exhibits low solar absorption and high infrared emissivity, thereby endowing wood with inherent radiative cooling capabilities [14]. By removing lignin to expose cellulose networks—with or without subsequent structural densification—the capacity of wood for thermal management [15,16,17,18,19,20,21] and flame resistance [22] may be simultaneously enhanced [23]. However, delignified wood remains largely limited to small-scale applications, whereas undelignified wood remains the dominant material in practical construction.

Unlike delignified or densified counterparts, which often require complex processing and may exhibit compromised structural integrity, undelignified wood retains its natural mechanical balance, cost-effectiveness, and compatibility with existing building practices [24]. Thus, enhancing the flame retardancy and thermal management capabilities of undelignified wood—without altering its native structure—holds significant practical value for widespread applications in construction. The development of undelignified wood with dual flame-retardant and thermal management properties remains insufficient [25]. Most often, only a single property (e.g., flame resistance or thermal management alone) is addressed, leaving a gap in solutions for mainstream undelignified wood that has not been met [26,27,28,29,30].

Current materials used for wood modification include metal oxides, polymers, and nanocomposites, each contributing specific functionalities to the treated material [31,32,33]. Silicon dioxide (SiO_2_) is particularly noteworthy: it exhibits low thermal conductivity, high thermal stability, and intrinsic flame-retardant properties, making it a theoretically ideal candidate effective for dual-performance modification [34,35]. However, applications for SiO_2_-modified wood have predominantly focused on hydrophobicity enhancement [36,37,38,39] or fungi resistance [40], with limited exploration of its potential for both flame retardancy and thermal management.

**Figure 1:**
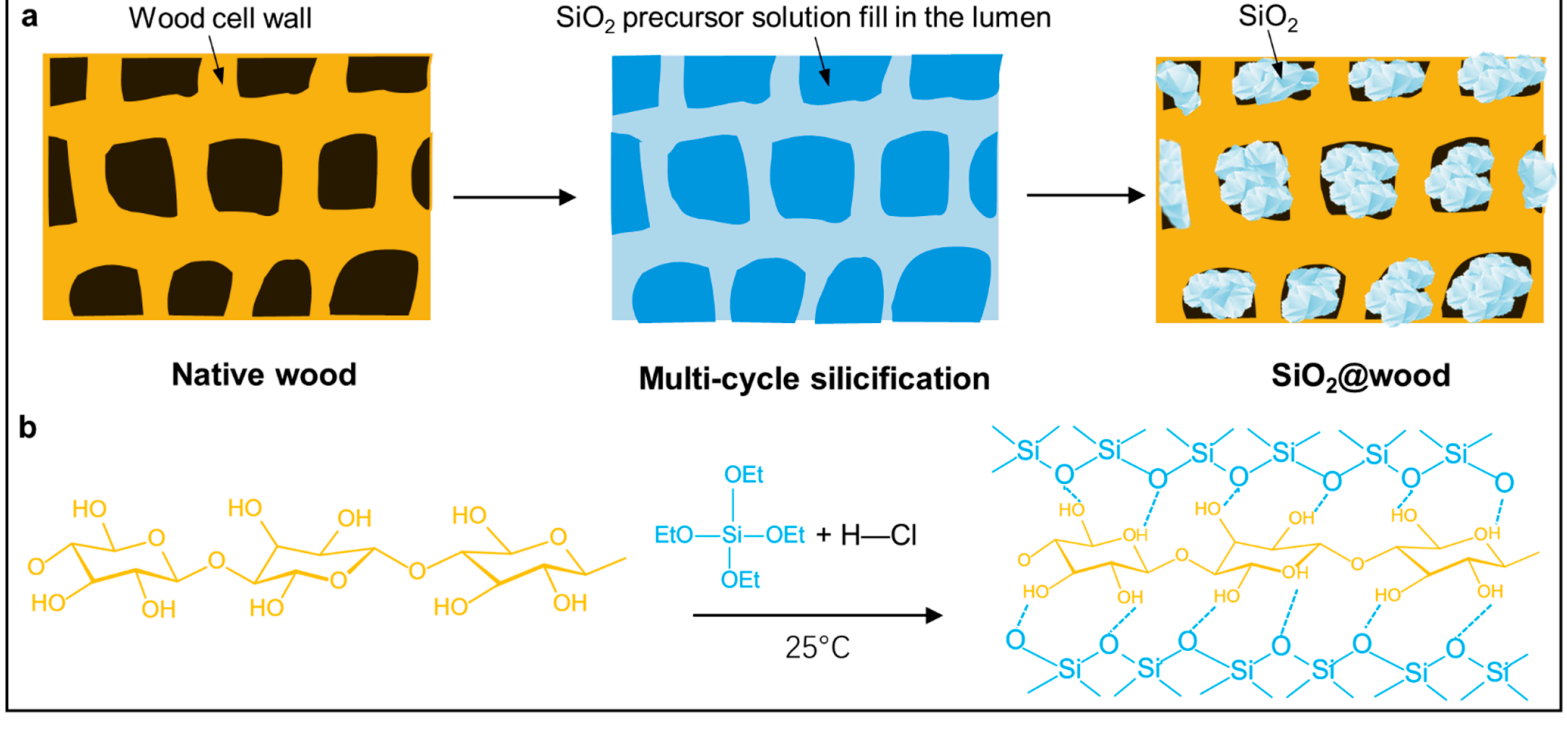
Illustration of (**a**) SiO_2_@wood fabrication and (**b**) chemical transformations.

A SiO_2_@wood with integrated flame-retardant and thermal management functionalities using undelignified wood via a multi-cycle silicification strategy has been developed (Figure 1a). Native wood (Norway spruce, *Picea abies*) was repeatedly impregnated with a Tetraethyl orthosilicate (TEOS)/ethanol/water solution (catalyzed by HCl) and dried, enabling stepwise infiltration of SiO_2_ precursors into wood lumens and subsequent hydrolysis–polycondensation. As illustrated in Figure 1b, SiO_2_ precursors undergo acid-catalyzed hydrolysis, which further condense with hydroxyl groups of wood cell walls via Si–O–C covalent bonding—this chemical interaction ensures stable immobilization of SiO_2_ within the wood structure. By leveraging the inherent properties of SiO_2_ and avoiding delignification, we seek to verify its ability to simultaneously enhance flame retardancy and thermal management in mainstream wood, providing a practical solution for high-performance, sustainable construction materials.

## 2. Materials and Methods

### 2.1. Materials

Radial sections of Norway spruce (*Picea abies*) with 10 cm × 10 cm × 1 cm dimensions (L × R × T) were used for SiO_2_@wood fabrication. Its moisture content was 13%, density was 440.8 kg/m^3^, and proportion of earlywood was 79%, exhibiting a fine texture and straight grain. Tetraethyl orthosilicate (TEOS, 99%) and absolute ethanol (C_2_H_5_OH, ≥99.7%), deionized water (DI water), and hydrochloric acid (HCl) were used without further purification.

### 2.2. Fabrication of SiO_2_@wood

SiO_2_@wood was synthesized via a repeated immersion–drying process to achieve uniform SiO_2_ infiltration into the wood structure. The protocol is detailed below. Firstly, a homogeneous transparent SiO_2_ precursor solution was prepared by mixing TEOS, DI water, and ethanol at a molar ratio of 1:0.46:2.92 (TEOS/water/ethanol) [40]. To initiate TEOS hydrolysis, 0.005 M HCl solution was added as a catalyst. Subsequently, the native wood was immersed in the SiO_2_ precursor solution at room temperature for 30 min. After that, SiO_2_@wood was dried at 60 °C for 120 min. This one-time impregnation procedure was repeated for 8 cycles.

### 2.3. Characterization

#### 2.3.1. Morphology

The morphological features and elemental distribution of native wood and SiO_2_@wood were characterized using a scanning electron microscope (SEM, Hitachi SU8000, Tokyo, Japan) equipped with an energy-dispersive X-ray spectrometer (EDX). Prior to SEM observation, samples were sputter-coated with a 10 nm thick Au layer using a CCU-010 sputter coater to enhance electrical conductivity. EDX elemental mapping was performed to analyze the distribution of carbon (C), oxygen (O), and silicon (Si) across the samples.

#### 2.3.2. Chemical Structure Characterization

Crystalline structures of wood samples were analyzed using an X-ray diffractometer (XRD, Rigaku Ultimate IV, Tokyo, Japan) with Cu Kα radiation (λ = 1.5406 Å). Measurements were conducted at 40 kV and 45 mA, with a 2θ range of 5°~80° and a scanning speed of 2°/min. Chemical functional groups were identified using Fourier transform infrared spectroscopy (FTIR, Thermo Fisher iS55, Waltham, Massachusetts, USA). Spectra were collected in ATR mode over the wavenumber range of 4000–400 cm^−1^ at a resolution of 4 cm^−1^, with 32 scans averaged per sample to minimize noise. Surface chemical composition and bonding states were analyzed using X-ray photoelectron spectroscopy (XPS, Thermo Fisher 250xi, Waltham, Massachusetts, USA). The full survey spectrum and high-resolution scans of C 1s, O 1s, and Si 2p were acquired under ultra-high vacuum (residual pressure ~1 × 10^−6^ Pa) with a pass energy of 55.0 eV. Peak fitting was performed using XPS Peak 4.1 software to determine chemical bonding configurations.

#### 2.3.3. Fire-Retardant Characterization

Thermal stability and char residue were evaluated using thermogravimetric analysis (TGA, TA Instruments, New Castle, DE, USA). Samples (~10 mg) were heated from 30 °C to 600 °C at a rate of 10 °C/min under a nitrogen flow rate of 60 mL/min. The tests were performed with three repetitions. Derivative thermogravimetric (DTG) curves were obtained to analyze the maximum decomposition rate and the corresponding temperature. Limiting oxygen index (LOI) values were measured in accordance with ASTM D2863 using an FTT 0077 instrument. Ten samples with the same dimensions (10 cm × 1.5 cm × 1 mm, L × R × T) were vertically mounted in the test chamber under a nitrogen atmosphere at 500 °C. Combustion behavior was visualized using a camera. Samples were exposed to the outer flame of an alcohol lamp, and ignition time, flame spread rate, and residual char morphology were recorded.

#### 2.3.4. Mechanical Characterization

Tensile mechanical properties of wood specimens with dimensions of 5 cm × 1.5 cm × 1 mm (L × R × T) were evaluated using a universal testing machine (CMT6104, Shenzhen, China). The tests were performed with five repetitions and conducted in accordance with the standard for tensile testing along the grain (GB/T 1927.14-2022).

#### 2.3.5. Thermal Management Characterization

Thermal conductivity was measured using a Hot Disk TPS 2500S thermal constant analyzer (NETZSCH LFA 457, Selb, Germany). Samples (1 cm × 1 cm × 1 mm, L × R × T) were tested at 25 °C under steady-state conditions, with three measurements per sample to ensure accuracy. Infrared reflectivity and transmittance were measured using a UV–vis–NIR spectrophotometer (Thermo Fisher Nicolet iS50, Waltham, Massachusetts, USA). Thermal insulation performance was evaluated using an FLIR Lepton infrared camera. Samples (5 cm × 1.5 cm × 1 mm, L × R × T) were placed on a heating plate set to 75 °C, and surface temperature distributions were recorded at 0, 1, 3, 5, and 8 s to analyze heat transfer dynamics.

## 3. Results and Discussion

### 3.1. Surface Morphology

**Figure 2:**
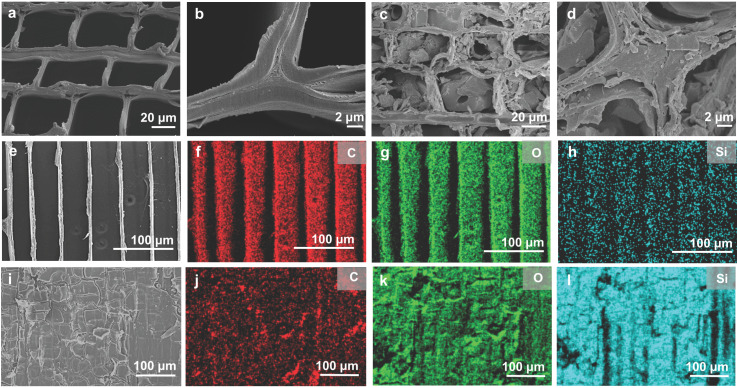
SEM and EDX characterizations of native wood and SiO_2_@wood. (**a**,**b**) Cross-section of native wood with different magnification. (**c**,**d**) Cross-section of SiO_2_@wood with different magnification. (**e**–**l**) Radial-section SEM images and EDX elemental mappings of (**e**–**h**) native wood and (**i**–**l**) SiO_2_@wood visualizing C (red), O (green), and Si (blue) distribution across the wood matrix.

The morphological characteristics of native wood and SiO_2_@wood were investigated via SEM and EDX to elucidate the distribution and infiltration of SiO_2_ within the wood structure (Figure 2). Native wood displays a typical hierarchical porous architecture, featuring open tracheid lumens and intact cell walls (Figure 2a,b). In contrast, after eight cycles of SiO_2_ precursor solution impregnation and drying, SiO_2_@wood exhibits a distinct structural transformation: the tracheid lumens are effectively filled with SiO_2_, forming a continuous and uniformly distributed network throughout the wood matrix (Figure 2c,d).

The radial–sectional view further confirms that SiO_2_ penetrates the entire wood structure and fills lumens (Figure 2e–l). EDX elemental mapping reveals critical compositional differences: for native wood, only carbon (C, red) and oxygen (O, green) signals are detected, consistent with its cellulose-, hemicellulose- and lignin-dominated composition (Figure 2e–h). Conversely, SiO_2_@wood shows uniform silicon (Si, blue) distribution across the wood matrix, with Si signals intensifying in regions corresponding to the filled lumens and infiltrated cell walls (Figure 2i–l). The Si distribution verifies that repeated immersion–drying cycles enable homogeneous SiO_2_ infiltration into the wood’s porous framework. This uniform infiltration of SiO_2_ into the natural porous framework of wood lays a critical structural foundation for enhancing material properties, as the filled lumens create a stable physical barrier within the wood matrix.

### 3.2. Chemical Composition Analysis

**Figure 3:**
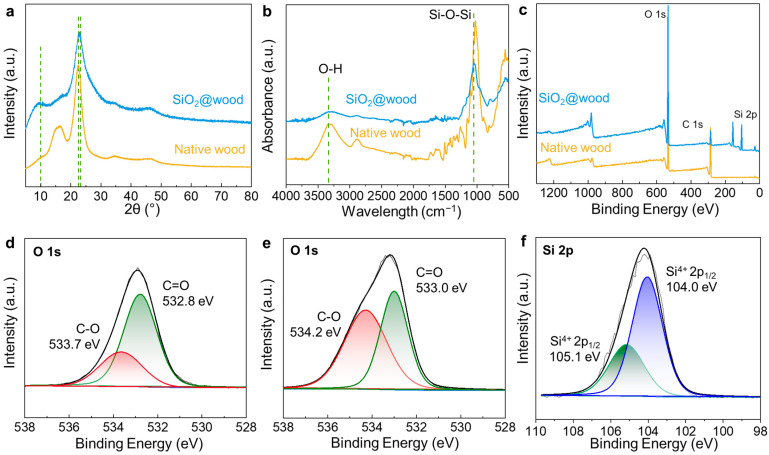
(**a**) XRD patterns of native wood and SiO_2_@wood. (**b**) FTIR spectra of native wood and SiO_2_@wood, showing variations in chemical functional groups. (**c**) XPS full spectrum of native wood and SiO_2_@wood. (**d**–**f**) High-resolution scan of O-1s for (**d**) native wood and (**e**) SiO_2_@wood, and (**f**) Si-2p for SiO_2_@wood. Grey lines represent the background baseline, and black lines represent the fitted curves.

Complementing the morphological observations, chemical characterization techniques including XRD, FTIR spectroscopy, and XPS were employed to unravel the phase composition and interfacial bonding in SiO_2_@wood, verifying the successful integration of SiO_2_ and its chemical interactions with the wood matrix. XRD patterns reveal that native wood exhibits a distinct crystalline peak at ~10° 2θ (corresponding to the 100 lattice plane of cellulose I) and a secondary peak at ~22° 2θ (101 lattice plane), collectively reflecting the ordered arrangement of cellulose microfibrils in the wood cell wall (Figure 3a). In stark contrast, SiO_2_@wood shows the ~10° peak is dramatically diminished and broadened, while a new broad diffraction halo centered at ~23° 2θ emerges—an intrinsic signature of amorphous SiO_2_ formed via TEOS hydrolysis and polycondensation. This amorphous nature avoids the brittleness associated with crystalline silica phases, preserving the wood matrix’s structural integrity. FTIR spectra further support these findings: native wood shows typical cellulose/hemicellulose/lignin peaks (e.g., O–H stretching at ~3400 cm^−1^, C–O vibrations at 1000–1200 cm^−1^), while SiO_2_@wood introduces a prominent new peak at ~1058 cm^−1^ (attributed to Si–O–Si asymmetric stretching) and a narrowed/weakened O–H band—collectively indicating silica incorporation and partial replacement of wood hydroxyl groups by Si–O–C bonds (Figure 3b).

XPS analysis quantifies these interactions: the survey spectrum of SiO_2_@wood introduces a distinct Si 2p signal (~103 eV, absent in native wood), confirming silica loading (Figure 3c–f). High-resolution O 1s spectra show that native wood’s C=O (532.8 eV) and C–O (533.7 eV) peaks shift to higher binding energies (533.0 eV and 534.2 eV, respectively) in SiO_2_@wood, consistent with electron density alterations from Si–O–C bond formation. Meanwhile, the Si 2p spectrum of SiO_2_@wood deconvolutes into Si^4+^ 2p_3_/_2_ (104.0 eV) and 2p_1_/_2_ (105.1 eV) peaks (2:1 area ratio), a hallmark of amorphous silica. Together, these chemical characterizations confirm that amorphous SiO_2_ infiltrates the wood structure, forms covalent Si–O–C linkages with cellulose, and modifies the chemical environment of both components—laying a critical foundation for SiO_2_@wood’s enhanced flame-retardant and thermal performance, as explored in subsequent sections.

### 3.3. Fire Retardancy and Mechanical Performance Evaluation

**Figure 4:**
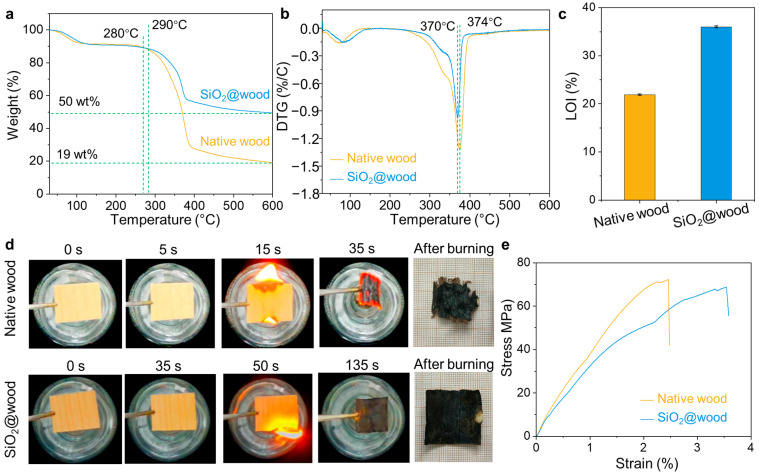
(**a**) TGA curves and (**b**) DTG profiles of native wood and SiO_2_@wood. (**c**) LOI values of native wood and SiO_2_@wood. (**d**) Time-resolved combustion images of native wood and SiO_2_@wood. (**e**) Stress−strain curves of native wood and SiO_2_@wood.

Building on the chemical structural insights into SiO_2_@wood, this section assesses its fire retardancy and mechanical performance through TGA, LOI, time-resolved combustion imaging, and tensile testing (Figure 4), elucidating how SiO_2_ incorporation modifies thermal degradation, flame resistance, and mechanical integrity for practical fire-safe and load-bearing applications. TGA and DTG analyses reveal distinct thermal stability improvements in SiO_22_@wood: Native wood initiates rapid weight loss at ~280 °C (Figure 4a) and shows a sharp DTG peak at ~374 °C (Figure 4b), corresponding to simultaneous decomposition of cellulose, hemicellulose, and lignin. In contrast, SiO_2_@wood delays significant weight loss onset to ~290 °C, exhibits a reduced DTG peak (maximum rate at ~370 °C), and retains a much higher residual char (~50 wt% at 600 °C vs. ~19 wt% for native wood). This confirms SiO_2_ acts as a thermal barrier—slowing the release of volatile fuel fragments by inhibiting heat feedback from potential combustion zones and promoting char formation, thus enhancing thermal stability. Further, the limiting oxygen index (LOI) quantifies flame resistance: native wood (LOI 21.9 ± 0.15%) behaves as a flammable material, while SiO_2_@wood achieves a substantially higher LOI of 36.0 ± 0.22% (Figure 4c), meeting fire-retardant criteria (LOI > 28%). Time-resolved combustion images (Figure 4d) visually corroborate this: native wood ignites within 15 s, burns violently by 35 s, and leaves fragmented char, whereas SiO_2_@wood delays ignition to 50 s, exhibits milder combustion through 135 s, and retains an intact, dense char layer post-burning—this SiO_2_-reinforced char functions as a critical insulation barrier: it inhibits heat feedback from the combustion zone to the underlying unburned material and reduces the rate of volatile fuel fragment formation, while physically isolating oxygen to suppress further degradation.

Notably, mechanical performance reveals a trade-off between strength and ductility optimized by SiO_2_ incorporation (Figure 4e). In the tensile test in the longitudinal direction, native wood exhibited an ultimate tensile stress of 72.33 ± 5.12 MPa with a strain of 2.46 ± 0.69%, which reflects the brittle failure of its porous structure. In contrast, SiO_2_@wood exhibits a slightly reduced ultimate tensile stress of 68.72 ± 6.23 MPa but a significantly enhanced strain of 3.5 ± 0.76%—which indicates improved ductility and toughness. This behavior arises from dual structural effects: (1) SiO_2_ infiltration fills lumens and reinforces cell walls, reducing defect-driven crack initiation; (2) Si–O–C bonding strengthens interfacial adhesion between silica and cellulose, enabling more uniform stress distribution and delayed crack propagation under tension. While native wood prioritizes high initial strength, SiO_2_@wood balances this with ~42% higher strain capacity—critical for applications requiring deformation tolerance (e.g., structural panels, insulation composites). Collectively, these results demonstrate that SiO_2_@wood balances fire safety and mechanical adaptability, advancing its potential for high-performance building and load-bearing scenarios.

### 3.4. Thermal Management Performance Assessment

**Figure 5:**
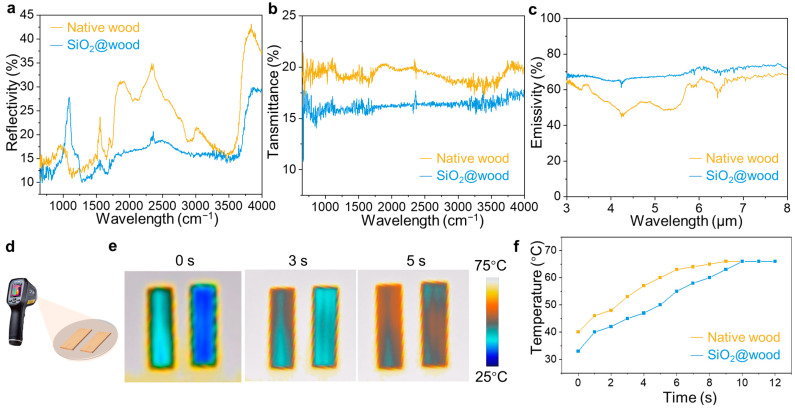
(**a**) Infrared reflectivity spectra of native wood and SiO_2_@wood. (**b**) Infrared transmittance spectra of native wood and SiO_2_@wood. (**c**) Infrared emissivity spectra of native wood and SiO_2_@wood. (**d**) Schematic of the thermal insulation testing setup. (**e**) Time-resolved thermal infrared images of native wood (left) and SiO_2_@wood (right). (**f**) Temperature–time plots of native wood and SiO_2_@wood.

The thermal management performance of SiO_2_@wood is critical for energy-efficient applications, which is systematically evaluated via thermal conductivity measurements, infrared transmittance spectra analysis, and time-resolved thermal imaging, with all samples standardized to 1 mm thickness to isolate structural effects on heat transfer. Thermal conductivity data reveal that native wood exhibits a higher thermal conductivity (0.078 ± 0.000047 W·m^−1^·K^−1^) compared to SiO_2_@wood (0.074 ± 0 W·m^−1^·K^−1^), attributed to SiO_2_ infiltration. Furthermore, SiO_2_@wood exhibits an advantageous low thermal conductivity in comparison with literature reports [41,42]. The amorphous silica networks fill wood lumens and pores, disrupting continuous heat conduction pathways and leveraging SiO_2_’s intrinsic low thermal conductivity to reduce overall conductive heat transfer. Infrared reflectivity spectra further demonstrate modified infrared radiation interaction (Figure 5a). SiO_2_@wood shows enhanced reflectivity light reflection in the 800–1350 cm^−1^ wavelength range, which is attributed to increased light scattering or interference effects induced by SiO_2_. However, such enhancement is less pronounced in other wavelength ranges, where the native wood substrate retains higher reflectivity. SiO_2_@wood exhibits lower transmittance (≤15% across most wavelengths) versus native wood (∼20% transmittance), indicating suppressed infrared radiation penetration through SiO_2_@wood (Figure 5b). Notably, as SiO_2_ is a high-emissivity material. Because Emissivity (%) = 100% − Reflectivity (%) − Transmittance (%), by integrating the reflectivity and transmittance data, Figure 5c shows SiO_2_@wood exhibits a significantly higher emissivity (peaking above 70%) in the 8–13 μm atmospheric infrared transparent window (a critical band for Earth’s radiative heat dissipation, where the atmosphere shows extremely weak absorption of radiation within this interval), whereas the native wood only demonstrates an emissivity of 40–60% in the same wavelength range. This result directly echoes the phenomena observed in thermal imaging. The thermal insulation testing setup (Figure 5d) and time-resolved thermal images (Figure 5e) provide direct visual validation: under identical heating, native wood (left) shows faster temperature rise (e.g., at 5 s, surface temperature reaches ∼60 °C) compared to SiO_2_@wood (right, ∼50 °C at 5 s), with SiO_2_@wood maintaining a lower equilibrium temperature even after 9 s. Specifically, temperature–time curves of native wood and SiO_2_@wood on a 75 °C heating plate are presented in Figure 5f. Within 0–10 s, SiO_2_@wood consistently exhibits a lower surface temperature than native wood, though their temperatures converge after 10 s. This delayed heating and reduced thermal response arise from the synergistic effects of reduced conductive heat transfer (via porous structure modification) and optimized radiative heat management. Collectively, these results demonstrate that SiO_2_@wood balances low thermal conductivity with tailored infrared radiation interaction, positioning it as a promising candidate for thermal insulation in energy-efficient building and industrial systems.

## 4. Conclusions

In conclusion, this study fabricates a silicified wood (SiO_2_@wood) via repeated SiO_2_ precursor solution infiltration and drying cycles. SiO_2_@wood shows superior fire retardancy: LOI rises to 36.0% (vs. 21.9% for native wood), with slower ignition (50 s vs. 15 s) and intact char formation. Mechanically, it balances properties—peak stress reaches ~68.72 MPa (slightly lower than native wood’s 72.33 MPa) but strain improves to 3.50% (vs. 2.46%). SiO_2_ uniformly fills wood lumens, reducing thermal conductivity to ~0.074 W·m^−1^·K^−1^ (vs. 0.078 W·m^−1^·K^−1^ for native wood) and enhancing emissivity. Thermal imaging confirms delayed heating (e.g., 5 s surface temp ~50 °C vs. 60 °C for native wood). However, it is crucial to acknowledge a limitation of the present work: the surface SiO_2_ layer exhibits restricted abrasion resistance, which necessitates the avoidance of friction-intensive scenarios to mitigate application limitations. With proper consideration of this constraint, SiO_2_@wood, leveraging its thermal insulation, fire resistance, and mechanical adaptability, emerges as a multifunctional candidate for energy-efficient green construction, advancing sustainable thermal management and fire-safe building solutions.

## Data Availability

The original contributions presented in this study are included in the article. Further inquiries can be directed to the corresponding authors.
